# Asymmetric dynamic coupling promotes alternative evolutionary pathways in an enzyme dimer

**DOI:** 10.1038/s41598-020-75772-5

**Published:** 2020-11-02

**Authors:** V. Ambrus, Gy. Hoffka, M. Fuxreiter

**Affiliations:** 1 MTA-DE Laboratory of Protein Dynamics, Department of Biochemistry and Molecular Biology, University of Debrecen, Debrecen, Hungary; 2 Department of Biomedical Sciences, University of Padova, Padua, Italy

**Keywords:** Biochemistry, Computational biology and bioinformatics, Evolution, Structural biology

## Abstract

The importance of dynamic factors in enzyme evolution is gaining recognition. Here we study how the evolution of a new enzymatic activity exploits conformational tinkering and demonstrate that conversion of a dimeric phosphotriesterase to an arylesterase in *Pseudomonas diminuta* is accompanied by structural divergence between the two subunits. Deviations in loop conformations increase with promiscuity, leading to functionally distinct states, while they decrease during specialisation for the new function. We show that opposite loop movements in the two subunits are due to a dynamic coupling with the dimer interface, the importance of which is also corroborated by the co-evolution of the loop and interface residues. These results illuminate how protein dynamics promotes conformational heterogeneity in a dimeric enzyme, leading to alternative evolutionary pathways for the emergence of a new function.

## Introduction

Proteins are dynamic entities, with a wide spectrum of conformational rearrangements linked to their functions^1,2^. Protein motions are crucial to optimise substrate interactions^3–5^, but their role in enzymatic catalysis is highly debated^6,7^. Here we were interested in the role of protein dynamics in the emergence of new enzymatic functions.

Evolution of enzymes usually involves multifunctional intermediates^8^, indicating that the active site can accommodate more than one substrate^9^. Increasing experimental data demonstrates that enzyme multi-functionality is a widespread phenomenon^8,10^. Thus, new functions can emerge, without considerable cost to the original activity^11^. Different active site geometries are optimized for alternative catalytic strategies^12^ or carry out distinct activities^13,14^. Specialisation for different activities also depends on the network context^10^, which modulates the populations of different conformational sub-states^15^.

Protein dynamics has also been proposed to contribute to evolvability^16–18^, as the conformational energy landscapes of enzymes need to be extensively remodelled upon the emergence of new functions^19,20^. However, the underlying mechanisms of how protein flexibility affects the emergence of the new and the decline of the original activity are yet to be elucidated^18,21^. The complete conversion of phosphotriesterase (PTE) to arylesterase (AE) via laboratory evolution^22^ provides a molecular record to study how dynamics contributes to a 10^9^-fold change in substrate selectivity. During the evolutionary pathway, the new activity improved in a gradually decreasing manner, indicating a slow convergence in function-optimisation, denoted as diminishing returns. The initial 10^2^–10^3^-fold increase in arylesterase activity was accompanied by a moderate decline in the phosphotriesterase activity^22^, leading to multi-functional intermediates.

**Figure 1:**
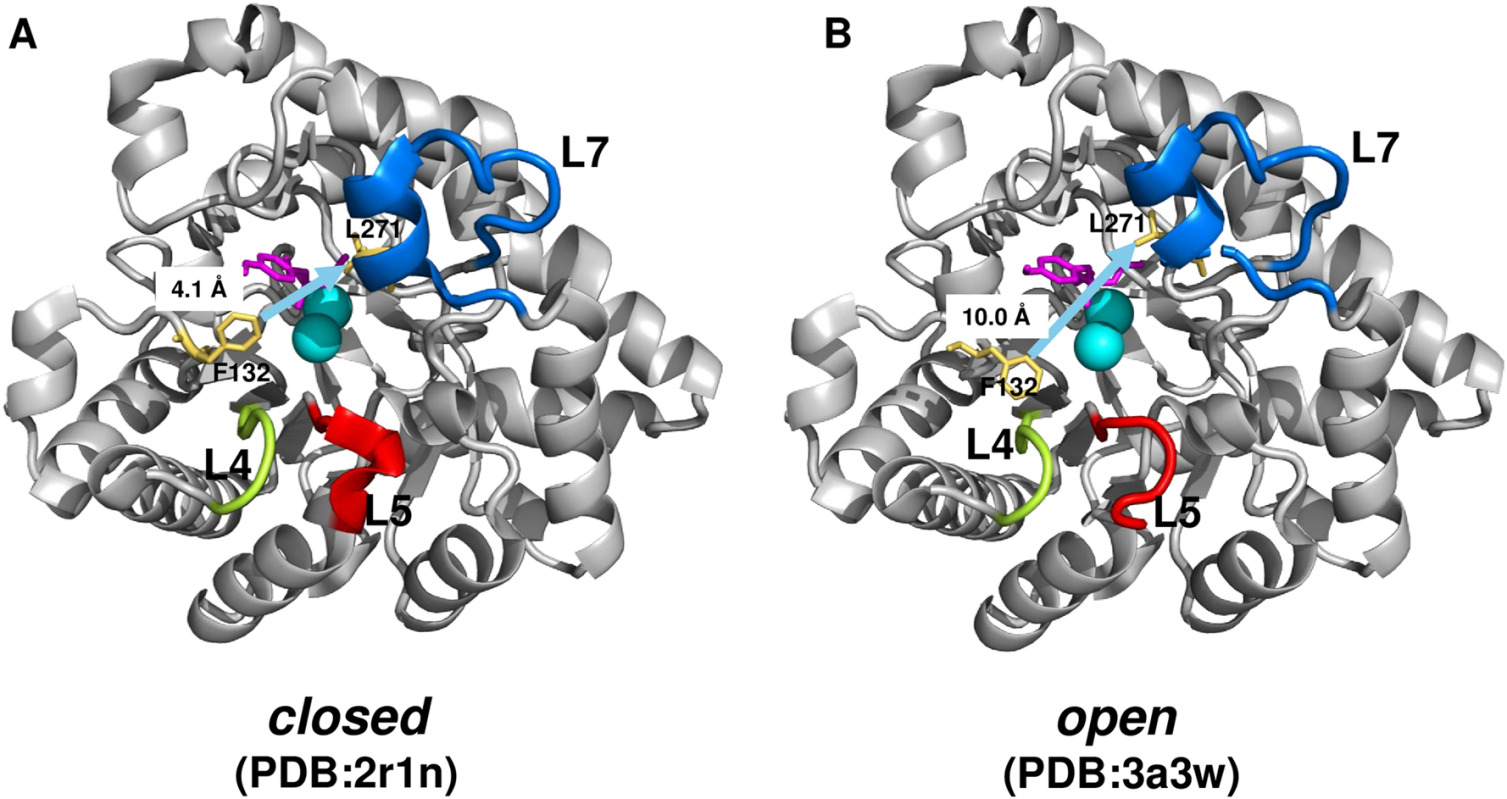
Conformational sub-states in phosphotriesterases with closed (**A**) and open (**B**) active sites. (**A**) The closed configuration (PDB: 2r1n) is optimal for the chemical step, but has a limited substrate access. (**B**) The open configuration (PDB:3a3w) is easily accessible for the substrate, but less optimised for phosphotriesterase conversion^12^. The F132–L271 distance (light blue arrow) shrinks from 10 to 4.1 Å from the open to the closed state. The loops are highlighted as **L4** (residues 171–175) lime, **L5** (residues 202–207) red, and **L7** (residues 258–274) blue. The substrate is magenta, the metal ions are displayed by cyan spheres.

Directed and natural evolution of bacterial phosphotriesterases revealed the existence of functionally distinct conformational sub-states^12^ (Fig. 1). The closed state is optimized for paraoxon hydrolysis, but requires considerable conformational rearrangements for substrate binding (Fig. 1A). In contrast, the open state is poorly organized for the chemical step, but it is optimal for the substrate access (Fig. 1B). Thus, efficient PTE hydrolysis requires an exchange between these two conformational sub-states to compromise between substrate diffusion and conversion^23^. During the PTE → AE conversion, three PTE loops exhibited substantial changes in dynamics^23^: **L4** (residues 171–175), **L5** (residues 202–207) and **L7** (residues 258–274) (Fig. 1). **L7** fluctuations were gradually frozen out to optimise the new function, while mobilities of **L4** and **L5** loops increased during the evolution^23^. Altered dynamics was proposed to shape the landscape of pre-existing catalytic states.

Here, we have investigated how the optimisation of functionally distinct conformational sub-states were synchronized in the enzyme dimer, as 6 out of the 26 mutations were located around the dimer interface (within 4.5 Å, Table S1). We observed that the overall change in dynamics, which was estimated by bioinformatics predictions from the sequences of the evolutionary intermediates, correlated to the decline in the original activity. However, we have observed considerable structural deviations between the two enzyme subunits during the PTE → AE trajectory, the magnitude of which has increased with promiscuity. We found that structural divergence was generated by the dynamic coupling between the loops and the dimer interface. Co-evolutionary analysis corroborated the functional link between the loop and interface residues. Taken together, our results highlight the role of protein dynamics via generating asymmetry between the enzyme subunits leading to alternative routes in the emergence of a new function.

## Results

### Changes in disorder correlate to the new activity

Protein disorder characterizes the preference of a sequence for a well-defined tertiary structure^24^. Disorder scores can be computed from the primary sequence and provide a coarse description of compactness and flexibility along the protein chain^25^, in accordance with NMR backbone dynamics^26^. Thus, disorder scores can used be to estimate the dynamic characteristics from a sequence in the absence of structural data.

**Figure 2:**
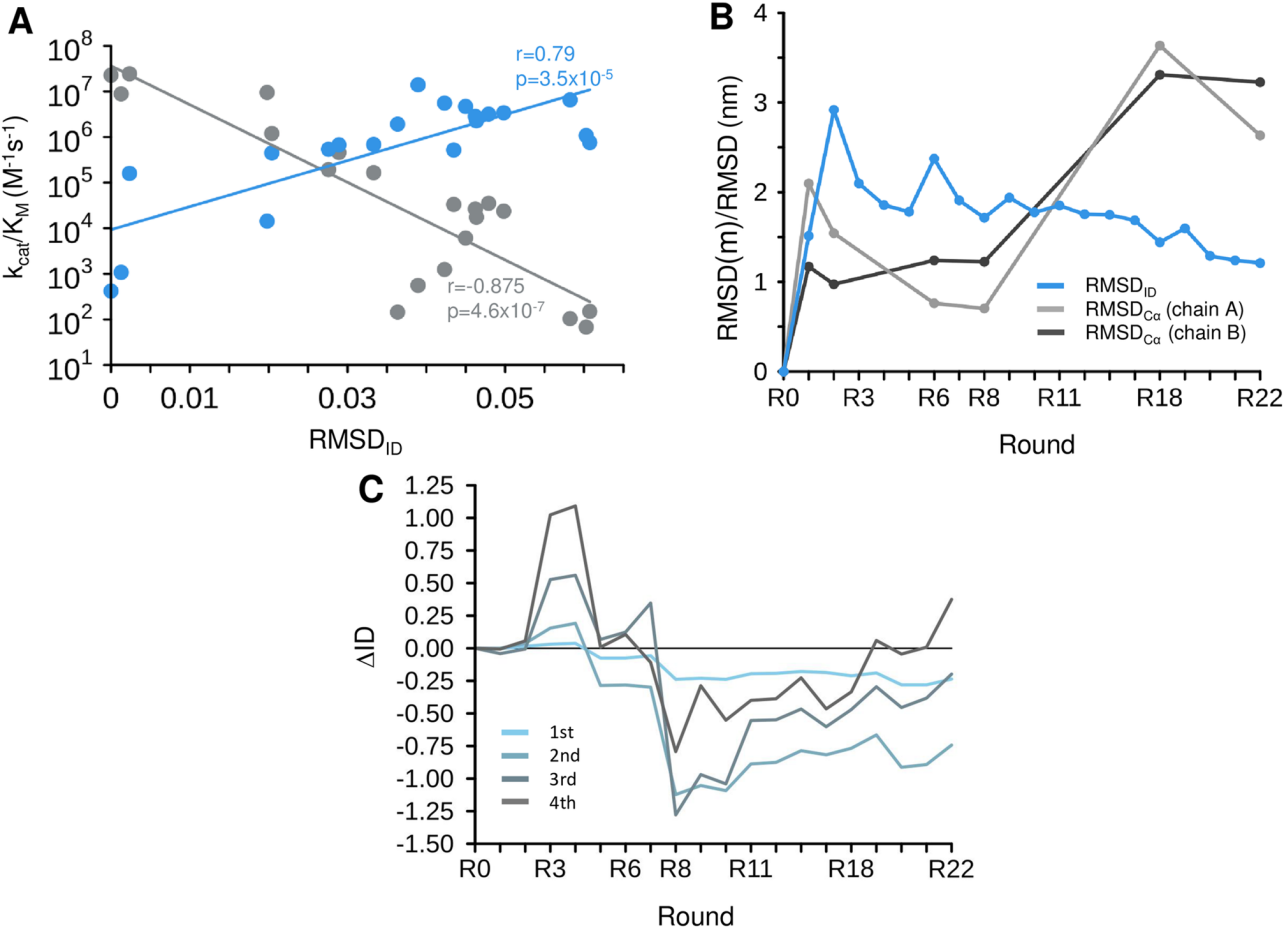
Changes in dynamics during the PTE → AE conversion. (**A**) Catalytic efficiencies (*k*_*cat*_*/K*_*M*_) of the original phosphotriesterase (grey) and of the new arylesterase (blue) activity of different evolutionary intermediates as a function of the change in disorder scores (*RMSD*_*ID*_) as defined by Eq. (1). The Pearson's r values and their statistical significances are displayed. (**B**) The impact of mutations on changes in dynamics and structure in different rounds of evolution. The ratio of changes in dynamics [${RMSD}_{ID }(\mathrm{m})/{RMSD}_{ID }(\mathrm{nm})$] of mutated and not mutated residues (blue) was defined by Eq. (3), the ratio of structural changes [${RMSD}_{C\alpha }(\mathrm{m})/{RMSD}_{C\alpha }(\mathrm{nm})$] was defined by Eq. (4), and was computed separately for the A (light grey) and B (dark grey) subunits. (**C**) Dynamics changes in coordination shells. The first shell comprises the active site (light blue), the second shell contains residues within 3.5 Å from the active site (dark blue), the third shell residues are within 3.5 Å from the second shell residues (light grey), and the fourth shell residues are the 3.5 Å from the third shell residues (dark grey). Changes in disorder were computed by Eq. (2).

We applied this approach to characterize dynamics in all evolutionary intermediates during the PTE → AE conversion, and extended the analysis to those variants whose structures have not been determined. Thus, we used the IUPred method^27^ to predict the disorder scores for the residues in all evolutionary intermediates^22^ (R1- R22) and compared them to those in the starting variant (R0) in a pair-wise manner (Methods). First, we computed the root-mean-square deviations of the disorder scores (RMSD_ID_), which characterised the magnitude of the changes in dynamics at a given point of the PTE → AE trajectory (Fig. 2A). We observed that such coarse-grained changes in dynamics correlated to the decrease of the original phosphotriesterase activity (Pearson’s correlations coefficient r = 0.87, significance p = 4.6 × 10^–7^). Correlation with the emerging arylesterase activity was weaker, but significant (Pearson’s correlations coefficient r = 0.79, significance p = 3.5 × 10^–5^) (Fig. 2A). These observations indicate that the functional switch PTE → AE requires systematic changes in protein motions.

Then, we aimed to probe the contributions of the mutations to the changes in dynamics. Thus, we determined the ratio of RMSD_ID_ values, which were computed separately for mutated and not-mutated residues (Methods). As the disordered scores were computed using long flanking windows (Methods), mutations could affect the scores of those residues which were far-lying in sequence. Our results showed that mutations had a larger impact on dynamics than residues which were not changed in evolution (Fig. 2B). In addition, we observed that in the beginning of the PTE → AE conversion mutations affected dynamics more than structure, while towards the end of the evolution this trend was reversed (Fig. 2B). With emerging arylesterase activity (R1–R6), the dynamic contributions of the mutated residues were considerably larger than during specialisation for the new function (R8–R22).

In addition, we observed that overall dynamics increased with promiscuity (R1–R6, Fig. 2C), while exhibited a sharp drop at the generalist state, where original and new activities were comparable. During optimisation of the arylesterase activity (R8–R22) protein dynamics gradually increased, most likely to facilitate structural adjustments for the new function.

Taken together, these results suggest that the emergence of the new function requires significant alterations to protein motions, which enable structural rearrangements for optimising the new activity. This is in accord with the proposed diminishing returns model of evolution^22^.

### Coupling between the loops and the interface

**Figure 3:**
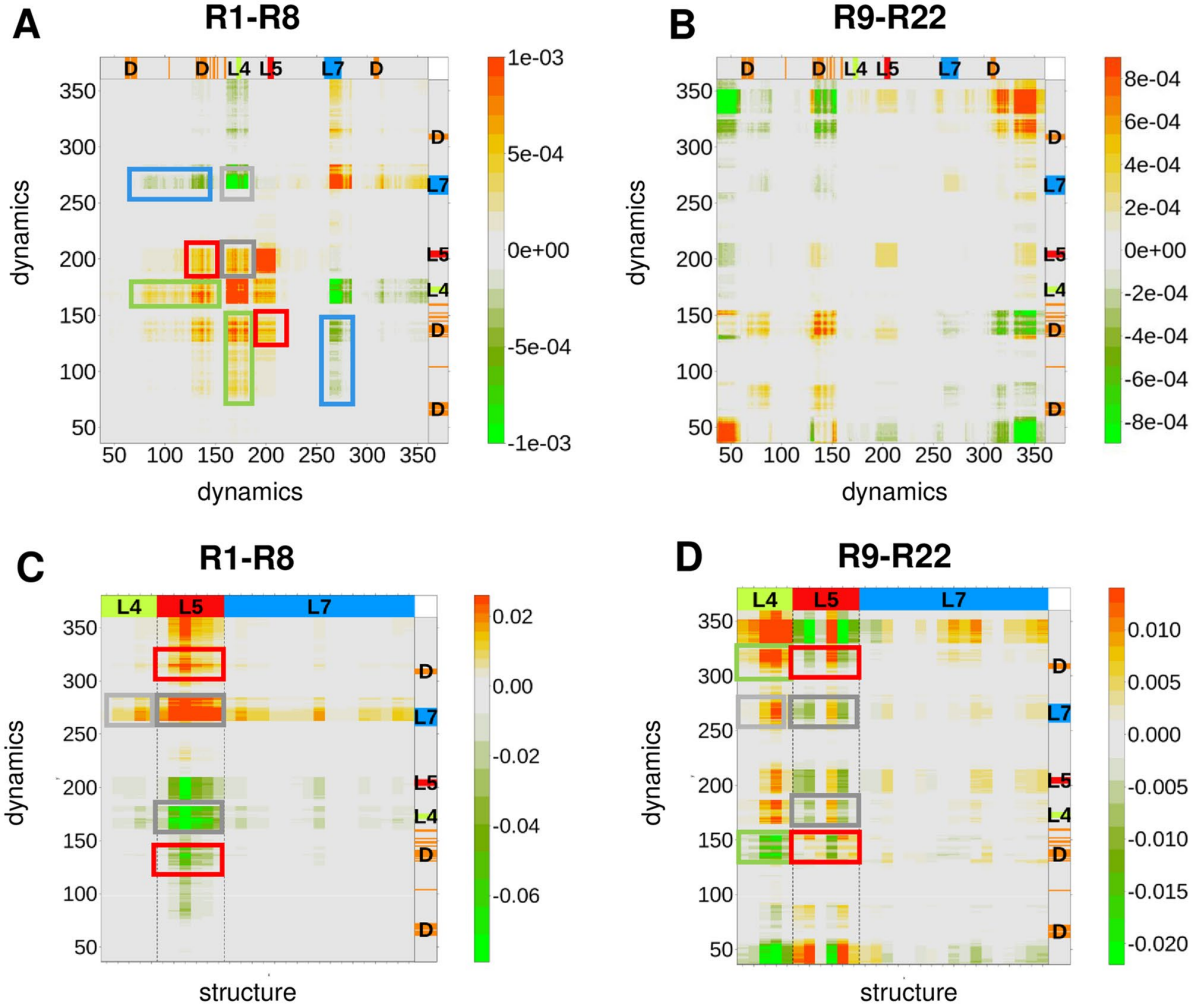
Covariance of dynamics and structure changes during the PTE → AE conversion. (**A**)–(**B**) Covariance matrices of changes in disorder scores as compared to the starting R0 variant (defined by Eq. 5), with emergence (**A**) and specialisation (**B**) for the new function. (**C**)–(**D**) Covariance matrices of deviations in disorder scores and structure from the starting R0 variant (defined by Eq. 6), with emergence (**C**) and specialisation (**D**) for the new function. The loops and the dimer interface are indicated on the side panels (**L4** green, **L5** red, **L7** blue, **D**-dimer interface wheat). Covariance between the loops and the dimer interface are marked by boxes coloured as the corresponding loops. Covariance between the loops are indicated by grey boxes. In the structure-dynamics covariance matrices only the loop structures are displayed for clarity.

In this section, we investigated how the changes in dynamics were synchronized in the two enzyme subunits during the evolution of the arylesterase function. Thus, we computed the covariance between the disorder scores throughout the PTE → AE conversion using all evolutionary intermediates (Methods) (Figs. 3A, 3B). We observed strong dynamic couplings in the first part of the PTE → AE conversion, when the new function emerged (Fig. 3A). The dynamics of the **L4** and **L5** loops in particular exhibited a strong positive covariance with increasing promiscuity (R1-R8), while those of the **L4** and **L7** loops were anti-correlated. This suggests that **L4** could serve as a clutch between **L5** and **L7** loops. The dimer interface exhibited a positive covariance with **L4** and **L5** dynamics, and a negative covariance with the **L7** loop (Fig. 3A). However, we did not observe significant covariance between the loops or with the dimer interface during specialisation for the new function (R9-R22), in accordance with the decrease in dynamical changes in this part of the trajectory (Fig. 3B).

We also analysed the covariance between conformation and dynamics in variants whose structures had been determined (R0, R1, R2, R6, R8, R18, R22, Table S3). During the emergence of the new function (R0-R8) we observed a strong, positive covariance between **L5** loop structure and **L7** dynamics, and a negative covariance between **L5** loop structure and **L4** dynamics (Fig. 3C). This suggests that changes in dynamics preceded structural rearrangements of the **L5** loop. In turn, structural changes of the **L5** loop were concomitant with the dynamical changes of the **L7** loop. **L5** structure was also strongly coupled to the dynamics of the dimeric interface (Fig. 3C). During the optimisation of the new function the structures of both **L5** and **L4** loops exhibited strong covariance with the interface (Fig. 3D). We observed opposite trends: while changes in dynamics of the interface induced conformational changes in the **L5** loop, they decreased structural changes of the **L4** loop (Fig. 3D). The **L7** loop structure did not exhibit pronounced coupling during the specialisation for the new function.

**Figure 4:**
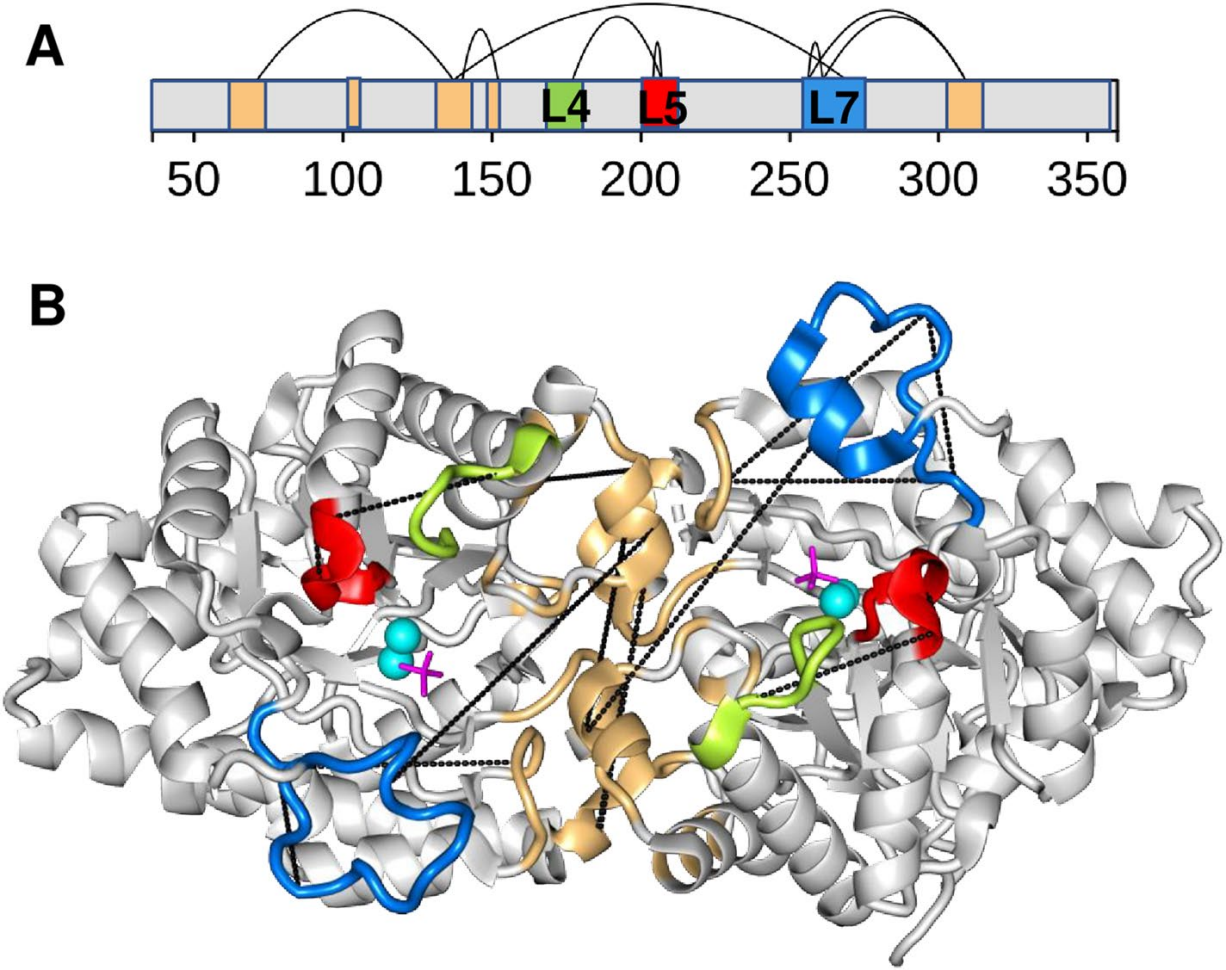
Co-evolving regions in *P. diminuta* phosphotriesterase. (**A**) Co-evolving residues in the **L4** (lime), **L5** (red), and **L7** (blue) loops and the dimer interface (wheat) are shown, as calculated with Gremlin webserver^28^. Co-evolutionary scores and significances are displayed in Table S2. (**B**) The structure of the R0 starting variant (PDB:4pcp) with the significant co-evolving residues displayed (black dashed lines). The loops are coloured as **L4** lime, **L5** red, and **L7** blue, and the dimer interface is shown in wheat. The substrate is magenta, the metal ions are displayed by cyan spheres.

We performed co-evolutionary analysis on the coupling between the loop and interface residues using the GREMLIN method^28^ (Methods, Fig. 4, Table S2). We found that residues of the **L7** loop and those of the dimer interface exhibit significant co-evolutionary signatures (Fig. 4). In addition, we observed a co-evolution between the **L4** and **L5** loops, as well as between the N- and C-terminal parts of the dimer interface. These results indicate that dynamical couplings between residues of the loops and the dimer interface, which drive the loop rearrangements, have been evolutionary conserved (Fig. 4).

Taken together, co-evolutionary results corroborated the functional importance of the dynamical coupling between the loops and the interface residues. Covariance between structure and dynamics indicates that dynamical couplings induce conformational rearrangements for the emergence of a new function.

### Functional promiscuity increases conformational divergence between the two subunits

Analysis of the structure-dynamics covariance indicated significant differences between the evolution of the two subunits (Figure S1). In the A subunit, the **L5** loop conformation exhibited positive covariance with the dynamics of the **L4** and **L7** loops and the dimer interface, while negative covariance was observed in the B subunit (Figure S1). These results suggest that dynamic variations, in particular of the dimer interface induced opposite changes in the conformation of the **L5** loop in the two subunits. Thus, we have compared the structures of the two subunits in all evolutionary variants with available structures (R0, R1, R2, R6, R8, R18, R22, Table S3). Indeed, we observed that the L5 loop exhibited remarkable deviations between the A and B subunits, especially in the generalist R6 and R8 states (Figure S2).

**Figure 5:**
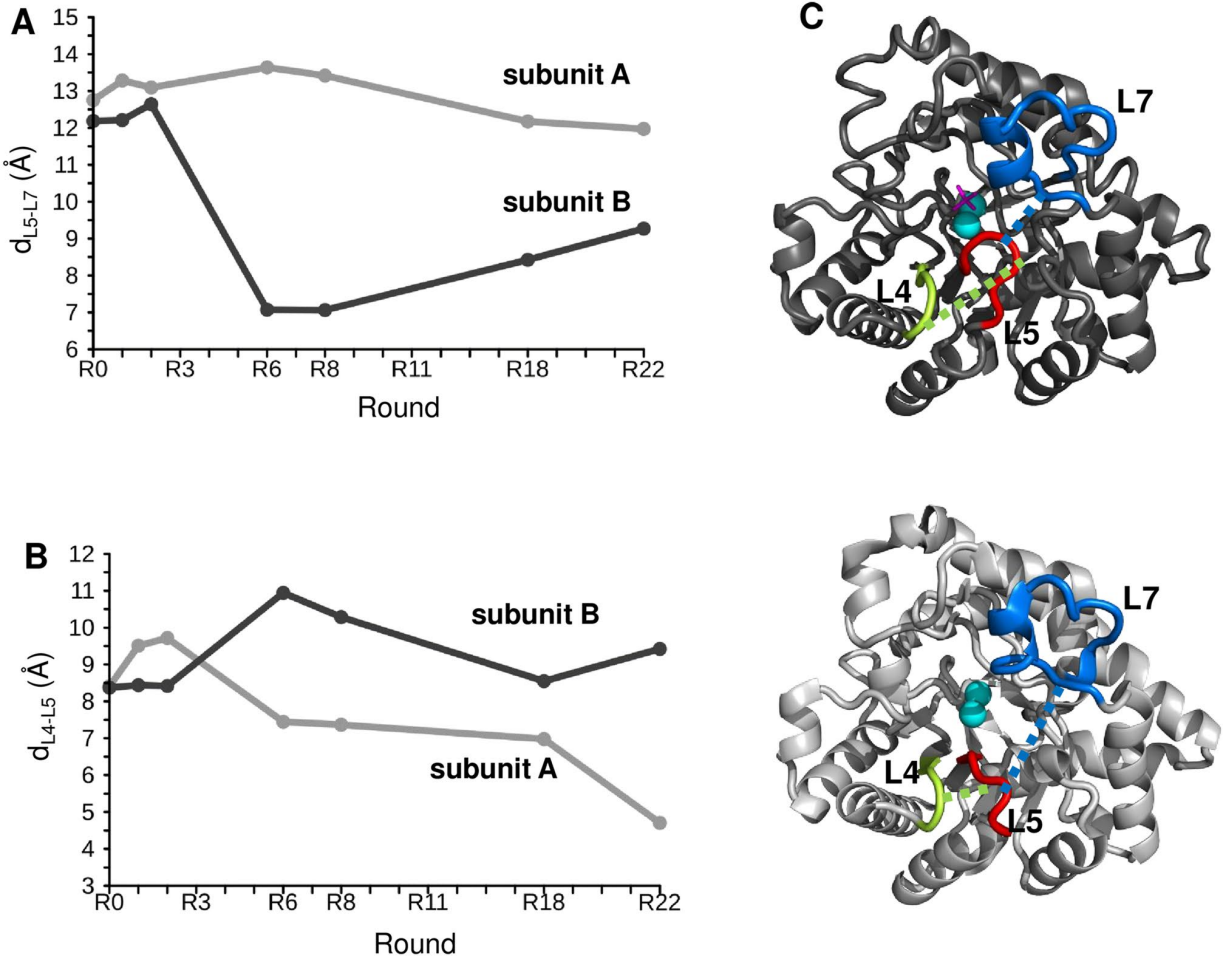
Loop distances during the PTE → AE conversion in the two subunits. (**A**) Variations in the distance of **L5** and **L7** loops defined as A/G204 Cα–G273 Cα in the **A** (light grey) and for the **B** (dark grey) subunits. (**B**) Variations in the distance of **L4** and **L5** loops defined as G174 Cα–S205 Cα in the **A** (light grey) and for the **B** (dark grey) subunits. (**C**) The representative loop positions and their distances in the structure of the B (dark, top) and A subunit (light, bottom) of the generalist R8 variant (PDB:4xay). The loops are coloured as **L4** lime, **L5** red, and **L7** blue; the **L5–L7** distance is displayed by a dashed blue line, and the **L5–L4** distance by a dashed green line. The substrate is magenta, the metal ions are displayed by cyan spheres.

We further analysed the deviations in **L5** conformation in the two subunits and focused on the distances between the loops (Fig. 5). We observed that in subunit B, the separation of the **L5** and **L7** loops (measured between A/G204 Cα and G273 Cα) decreased substantially during the PTE → AE trajectory, in particular around the generalist R6 and R8 states (Fig. 5, Table S4). In parallel, the **L5** loop moved away from **L4** in subunit B (measured between G174 Cα and S205 Cα). In subunit A, we observed the opposite trend, the **L5**–**L4** separation decreased, while the distance between the **L5** and **L7** loops increased slightly with increasing promiscuity (Fig. 5, Table S4). The variations in the **L5**–**L7** distance rationalise why stabilisation of **L7** is coupled to destabilisation of **L5**^23^.

Taken together, we observed an asymmetry between the dynamical couplings between the loops and the dimer interface, reflecting the opposite movements of the loops in the two subunits.

### Alternative evolutionary pathways in the two subunits

We investigated the functional consequences of the different loop positions in the two subunits. First, we compared the two subunits in the generalist R8 intermediate (PDB:4xay) to the structures representing the open (PDB: 3a3w) and closed (PDB:2r1n) states of PTE^12^ (Figure S3). These structures exhibit some differences in the conformation of the **L7** loop, which considerably resize the active size. Although the **L5** has a helical conformation in the closed state and is a loop in the open state, its separation from the **L4** and **L7** loops were rather similar (Figure S3). The position of the **L5** loop in the open and closed PTE structures was in between those in the two subunits of the generalist R8 state (Figure S3). These observations indicate that different loop conformations may not be related to the optimisation of substrate binding versus the chemical step.

To obtain further insights into the different loop architectures, we assembled PTE structures from the Protein Data Bank, which represented snapshots of different evolutionary pathways in different organisms. We compared these structures to those of the R6 (PDB: 4xag) and R8 intermediates (PDB:4xay) and analysed the representative distances (**L5**-**L7**: A/G204 Cα–G273 Cα; **L5**-**L4**: G174 Cα–S205 Cα) between the loops. From the dataset of 160 PTE-related structures we selected 19 structures which had kinetic data on phosphotriesterase or promiscuous activities (Table S5). We observed that enzymes with loop distances similar to the G chain (B subunit) R6 and R8 intermediates, also had considerable arylesterase activity (Table S5), or hydrolysed other substrates (not shown). In those enzymes structures which resemble the loop conformations in the A subunit primarily functioned as phosphotriesterases and were not observed to cleave other substrates. These results suggest that the two subunits of the enzyme dimer have different substrate preferences and catalytic activities in the generalist states. The A subunit is likely specialized on paraoxon hydrolysis, whereas the B subunit is promiscuous, with considerable arylesterase activity.

Taken together, the comparative structural analysis of the subunit conformations to different PTE enzymes indicates that the deviation between the loop architecture has a functional relevance. While the loop conformations in the A subunit are compatible with paraoxon hydrolysis, those in the B subunit have increased preference for arylester hydrolysis. These results implicate that the evolution in the two subunits follows different pathways.

## Discussion

The role of conformational heterogeneity in enzymatic catalysis^29^ as well as in the emergence of new protein functions is being increasingly recognised^5^. Within this framework, mutations modulate the balance between different conformations, which are compatible with alternative functions^16^. This sheds light on the role of protein dynamics in protein evolution via modulating conformational ensembles by distant amino acid replacements. Changes in low-frequency structural fluctuations may also facilitate the emergence of new functions^7,30^, but their interpretation is far from being trivial^6^. Directed evolution of PTE from *Pseudomonas diminuta* to arylesterase^22^ exemplifies how protein evolution engineers protein motions via freezing out fluctuations, which are unproductive for the emerging function.

Our results highlight a novel angle of how catalytic promiscuity stems from conformational heterogeneity. We have found that structural divergence between the two subunits of the *Pseudomonas diminuta* phosphotriesterase accompanies the evolution of the arylesterase function. Deviation in loop conformations between the two subunits increases with promiscuity. We have shown that dynamic couplings between the loops and the dimer interface, especially during the emergence of the new function, are responsible for the structural divergence (Fig. 3). These results rationalise the role of mutations, which are located at the dimer interface distant from the catalytic centre (Table S1). The functional importance of these dynamic couplings was further corroborated by co-evolutionary data (Fig. 4).

Comparative analysis using a set of PTE enzymes with structures and kinetic data available indicated that the different loop conformations represent functionally distinct states (Table S5). While the loop distances in the A subunit are compatible with phosphotriesterase hydrolysis, in the B subunit (G chain in R6 and R8 intermediates) they are similar to structures with promiscuous activities. Thus, we may conclude that the conversion to arylesterase in the B subunit precedes that of the A subunit. The considerable PTE activity of the A subunit, even at the end of the evolution, was beneficial for the reverse pathway from arylesterase to phosphotriesterase^31^. Quantifying the difference between the activities of the individual subunits requires state-of-the art QM/MM methods^32^ because of the metal ions involved in catalysis^33^ and the heterogeneity of the system^34,35^, which is being carried out in our laboratory using different simulation methods^32^.

Taken together, our results indicate that the conversion of PTE to arylesterase is not synchronized in the two subunits of the enzyme dimer. This is analogous to gene duplication; one copy may undergo significant sequence changes, while the other is more restricted to the original function^36^. Thus, we propose that dimerisation can promote alternative pathways in enzyme evolution via structural divergence due to asymmetric dynamic couplings.

## Methods

### Disorder predictions

The preference for a well-defined versus a disordered structure was computed using the IUPred program^27^. Disorder scores correlate to NMR backbone dynamical parameters^26^, thus they can be used to estimate coarse-grained dynamics at the residue level. We used the sequences of all the intermediates of the PTE → AE conversion^22,31^ as an input for the IUPred long algorithm to predict the disorder scores. We have also performed predictions using the short version of the IUPred program, the Espritz NMR^37^ and Dynamine^38^ methods. The results were found to be consistent with each other.

### Characterisation of changes in dynamics

The following quantities were used to assess the impact of evolution on dynamics. The magnitude of the overall changes in dynamics was characterised by the root-mean-square deviation of the ID scores from the starting variant:

1 $${RMSD}_{ID}(X)=\sqrt{\frac{\sum_{i=1}^{n}{({ID}_{i}^{X}-{ID}_{i}^{R0})}^{2}}{n}}$$

where ${RMSD}_{ID}(X)$ is the root-mean square deviation of the disorder scores in the X evolutionary intermediate from the starting (R0) sequence. ${ID}_{i}^{X}$ and ${ID}_{i}^{R0}$ are the ID scores of the *i*th residue in the X and R0 variant. The RMSD is computed from the scores of all residues, *n* is the number of residues in the protein.

The change in dynamics in given coordination shells relative to the starting variant was characterised by the sum of the differences between the ID scores of the corresponding residues.

2 $$\Delta {ID}_{j}(X)=\sum_{i=1}^{m}{({ID}_{i}^{X}-{ID}_{i}^{R0})}$$

where ${ID}_{i}^{X}$ and ${ID}_{i}^{R0}$ are the ID scores of the *i*th residue in the X and R0 variant. *m* is the number of residues in the given (*j*) coordination shell.

The impact of mutations on dynamics in the X variant was quantified by the ratio of the ${RMSD}_{ID}(X)$ values of the mutated (*m*) and not mutated (*nm*) residues:

3 $${RMSD}_{ID }(\mathrm{m})/{RMSD}_{ID }(\mathrm{nm})= \sqrt{\frac{\sum_{i=1}^{m}{({ID}_{i}^{X}-{ID}_{i}^{R0})}^{2}}{m}} / \sqrt{\frac{\sum_{j=1}^{nm}{({ID}_{j}^{X}-{ID}_{j}^{R0})}^{2}}{nm}}$$

where ${RMSD}_{ID}(X)$ is the root-mean square deviation of the disorder scores in the X evolutionary intermediate from the starting (R0) sequence. ${ID}_{i}^{X}$ and ${ID}_{i}^{R0}$ are the ID scores of residue *i* in the X intermediate and the R0 starting variant. Residues were grouped based on whether they were mutated or not in the given round of the PTE → AE conversion. *m* is the number of mutated, *nm* is the number of not mutated residues.

**Structure analysis.** Structures of the evolutionary intermediates were assembled from the Protein Data Bank (Table S3). We noticed a considerable difference between the number and occupancy of the metal ions (Table S3) which are involved in the catalytic step. Structures of the evolutionary intermediates were compared to those of the starting variant (PDB:4pcp). The PTEs with open (PDB: 3a3w) and closed (PDB: 2r1n) active sites (Fig. 1) were derived from earlier studies^12^. PTE structures with different evolutionary pathways were assembled based on sequence similarity (Table S5).

The impact of mutations on structure in the X variant was quantified by the ratio of the ${RMSD}_{C\alpha }(X)$ values of the mutated (*m*) and not mutated (*nm*) residues:

4 $${RMSD}_{C\alpha }(\mathrm{m})/{RMSD}_{C\alpha }(\mathrm{nm})= \sqrt{\frac{\sum_{i=1}^{m}{({P}_{i,C\alpha }^{X}-{P}_{i,C\alpha }^{R0})}^{2}}{m}} / \sqrt{\frac{\sum_{j=1}^{nm}{({P}_{j,C\alpha }^{X}-{P}_{j,C\alpha }^{R0})}^{2}}{nm}}$$

where ${RMSD}_{C\alpha }(X)$ is the root-mean square deviation of the $C\alpha$ positions in the X evolutionary intermediate from the starting (R0) structure. ${P}_{i,C\alpha }^{X}$ and ${P}_{i,C\alpha }^{R0}$ are the positions of the $C\alpha$ atom of residue *i* in the X intermediate and the R0 starting variant. Residues were grouped based on whether they were mutated or not in the given round of the PTE → AE conversion. *m* is the number of mutated, *nm* is the number of not mutated residues.

In the analysis of loop distances, the corresponding residue numbers in the PDB structures were identified based on sequence alignment to the wild type PTE sequence.

### Covariance analysis

The covariance of changes in dynamics was defined as:

5 $${K}_{{ID}_{i},{ID}_{j}}= cov\left[{\Delta ID}_{i},{\Delta ID}_{j}\right]=cov\left[{(ID}_{i}-{ID}_{0}),{(ID}_{j}-{ID}_{0})\right]$$

where ${\Delta ID}_{i}$ and ${\Delta ID}_{j}$ are the differences in disorder scores of residues *i* and *j* from the starting R0 variant.

The covariance of structure and dynamics changes were defined as:

6 $${K}_{{ID}_{i},{C\alpha }_{j}}= cov\left[{\Delta ID}_{i},{\Delta C\alpha }_{j}\right]=cov\left[{(ID}_{i}-{ID}_{0}),{(C\alpha }_{j}-{C\alpha }_{0})\right]$$

where ${\Delta ID}_{i}$ is the difference in disorder scores of residue *i* from the starting R0 variant. ${\Delta C\alpha }_{j}$ is the difference in Cα of residue *j* from the starting R0 variant.

Covariance matrices were calculated using the R software package (https://www.R-project.org/).

### Co-evolution analysis

Co-evolving residue pairs were identified with GREMLIN webserver^28^ using the wild type PTE^22^ sequence. The GREMLIN method is based on a multiple sequence alignment (MSA) by HHblits^39^ with homologue sequences > 90% identity, > 75% coverage and < 25% gaps in MSA. The PTE (A0A060GYS1) co-evolutionary analysis was performed using 819 sequences. Co-evolving amino acids were defined as residue pairs above a scaled score of 1 (Table S2).

## Supplementary information

Supplementary information.

Supplementary Table.

## References

[1] Henzler-Wildman K, Kern D (2007). Dynamic personalities of proteins. Nature.

[2] Lewandowski JR, Halse ME, Blackledge M, Emsley L (2015). Protein dynamics. Direct observation of hierarchical protein dynamics. Science.

[3] Privett HK (2012). Iterative approach to computational enzyme design. Proc. Natl. Acad. Sci. USA.

[4] Blomberg R (2013). Precision is essential for efficient catalysis in an evolved Kemp eliminase. Nature.

[5] Petrovic, D., Risso, V. A., Kamerlin, S. C. L. & Sanchez-Ruiz, J. M. Conformational dynamics and enzyme evolution. *J. R. Soc. Interface* **15** (2018). 10.1098/rsif.2018.0330 PMC6073641 30021929

[6] Kamerlin SC, Warshel A (2010). At the dawn of the 21st century: Is dynamics the missing link for understanding enzyme catalysis?. Proteins.

[7] Bhabha G (2011). A dynamic knockout reveals that conformational fluctuations influence the chemical step of enzyme catalysis. Science.

[8] Copley SD (2017). Shining a light on enzyme promiscuity. Curr. Opin. Struct. Biol..

[9] Khersonsky O, Tawfik DS (2010). Enzyme promiscuity: a mechanistic and evolutionary perspective. Annu. Rev. Biochem..

[10] Nam H (2012). Network context and selection in the evolution to enzyme specificity. Science.

[11] Aharoni A (2005). The 'evolvability' of promiscuous protein functions. Nat. Genet..

[12] Jackson CJ (2009). Conformational sampling, catalysis, and evolution of the bacterial phosphotriesterase. Proc. Natl. Acad. Sci. USA.

[13] Allali-Hassani A (2007). Structural and chemical profiling of the human cytosolic sulfotransferases. PLoS Biol..

[14] Hong NS (2018). The evolution of multiple active site configurations in a designed enzyme. Nat. Commun..

[15] Fuxreiter M (2018). Towards a stochastic paradigm: from fuzzy ensembles to cellular functions. Molecules.

[16] James LC, Tawfik DS (2003). Conformational diversity and protein evolution—a 60-year-old hypothesis revisited. Trends Biochem. Sci..

[17] Tokuriki N, Tawfik DS (2009). Protein dynamism and evolvability. Science.

[18] Campbell EC (2018). Laboratory evolution of protein conformational dynamics. Curr. Opin. Struct. Biol..

[19] Zou T, Risso VA, Gavira JA, Sanchez-Ruiz JM, Ozkan SB (2015). Evolution of conformational dynamics determines the conversion of a promiscuous generalist into a specialist enzyme. Mol. Biol. Evol..

[20] Colin PY (2015). Ultrahigh-throughput discovery of promiscuous enzymes by picodroplet functional metagenomics. Nat. Commun..

[21] Pabis A, Risso VA, Sanchez-Ruiz JM, Kamerlin SC (2018). Cooperativity and flexibility in enzyme evolution. Curr. Opin. Struct. Biol..

[22] Tokuriki N (2012). Diminishing returns and tradeoffs constrain the laboratory optimization of an enzyme. Nat. Commun..

[23] Campbell E (2016). The role of protein dynamics in the evolution of new enzyme function. Nat. Chem. Biol..

[24] van der Lee R (2014). Classification of intrinsically disordered regions and proteins. Chem. Rev..

[25] Sormanni P (2017). Simultaneous quantification of protein order and disorder. Nat. Chem. Biol..

[26] Daughdrill GW, Borcherds WM, Wu H (2011). Disorder predictors also predict backbone dynamics for a family of disordered proteins. PLoS ONE.

[27] Dosztanyi Z, Csizmok V, Tompa P, Simon I (2005). The pairwise energy content estimated from amino acid composition discriminates between folded and intrinsically unstructured proteins. J. Mol. Biol..

[28] Kamisetty H, Ovchinnikov S, Baker D (2013). Assessing the utility of coevolution-based residue-residue contact predictions in a sequence- and structure-rich era. Proc. Natl. Acad. Sci. USA.

[29] Lu HP, Xun L, Xie XS (1998). Single-molecule enzymatic dynamics. Science.

[30] Klinman JP, Kohen A (2014). Evolutionary aspects of enzyme dynamics. J. Biol. Chem..

[31] Kaltenbach M, Jackson CJ, Campbell EC, Hollfelder F, Tokuriki N (2015). Reverse evolution leads to genotypic incompatibility despite functional and active site convergence. Elife.

[32] Sousa SF (2017). Application of quantum mechanics/molecular mechanics methods in the study of enzymatic reaction mechanisms. WIREs Comput. Mol. Sci..

[33] Berta D, Buigues PJ, Badaoui M, Rosta E (2020). Cations in motion: QM/MM studies of the dynamic and electrostatic roles of H(+) and Mg(2+) ions in enzyme reactions. Curr. Opin. Struct. Biol..

[34] Sanchez-Martinez M, Marcos E, Tauler R, Field M, Crehuet R (2013). Conformational compression and barrier height heterogeneity in the N-acetylglutamate kinase. J. Phys. Chem. B.

[35] Kots ED, Lushchekina SV, Varfolomeev SD, Nemukhin AV (2017). Role of protein dimeric interface in allosteric inhibition of N-acetyl-aspartate hydrolysis by human aspartoacylase. J. Chem. Inf. Model..

[36] Soskine M, Tawfik DS (2010). Mutational effects and the evolution of new protein functions. Nat Rev Genet.

[37] Walsh I, Martin AJ, Di Domenico T, Tosatto SC (2012). ESpritz: accurate and fast prediction of protein disorder. Bioinformatics.

[38] Cilia E, Pancsa R, Tompa P, Lenaerts T, Vranken WF (2013). From protein sequence to dynamics and disorder with DynaMine. Nat. Commun..

[39] Remmert M, Biegert A, Hauser A, Soding J (2011). HHblits: lightning-fast iterative protein sequence searching by HMM-HMM alignment. Nat. Methods.

