# Supplementary material for: Asymmetric dynamic coupling promotes alternative evolutionary pathways in an enzyme dimer
Source: Sci Rep. 2020 Nov 2;10:18866. doi: 10.1038/s41598-020-75772-5 (PMC7608688; doi:10.1038/s41598-020-75772-5)
Supplement: Supplementary file 1 — Supplementary information. [file 41598_2020_75772_MOESM1_ESM.docx]

**Asymmetric dynamic coupling promotes alternative evolutionary pathways in an enzyme dimer**

V. Ambrus^1^, Gy. Hoffka^1^, M. Fuxreiter^1*^

*^1^MTA-DE Laboratory of Protein Dynamics, Department of Biochemistry and Molecular Biology, University of Debrecen, Hungary*

**Table S1 Distances of the mutated residues from the loops (L4, L5, L6) and the dimer interface (DI) in the R0 variant** (PDB: 4pcp). Loops were defined as **L4** (171-175 aa), **L5** (202-207 aa), **L7** (258-274 aa). The dimer interface contained residues within 4.5 Å of the other subunit (61-72; 104; 131-141; 145; 148-149; 152; 159-160; 307-311 aa). The active site (AS) comprised the zinc coordinating residues (55; 57; 169; 201; 230; 301 aa). The minimum distance between the Cα of the mutated residue and Cα of the other residues in the given region is displayed (Å).

| **subunit A** | | | | | | **subunit G** | | | | | |
| --- | --- | --- | --- | --- | --- | --- | --- | --- | --- | --- | --- |
| **AA** | **AS** | **DI** | **L4** | **L5** | **L7** | **AA** | **AS** | **DI** | **L4** | **L5** | **L7** |
| **H254R** | 5.85 | 16.73 | 15.07 | 10.99 | 3.80 | **H254R** | 5.76 | 16.77 | 15.32 | 11.24 | 3.81 |
| **D233E** | 5.85 | 17.87 | 12.92 | 5.52 | 8.17 | **D233E** | 5.83 | 17.83 | 13.03 | 5.57 | 8.23 |
| **F306I** | 9.32 | 3.79 | 17.75 | 19.46 | 12.59 | **F306I** | 9.29 | 3.83 | 17.70 | 19.15 | 12.35 |
| **I274S** | 14.97 | 19.56 | 20.07 | 13.19 | 3.81 | **I274S** | 14.77 | 18.13 | 20.00 | 13.55 | 3.79 |
| **T172I** | 6.33 | 7.11 | 0.00 | 5.86 | 15.99 | **T172I** | 6.38 | 6.95 | 0.00 | 5.98 | 16.34 |
| **S269T** | 15.65 | 12.98 | 17.91 | 14.53 | 0.00 | **S269T** | 16.16 | 12.13 | 18.97 | 15.45 | 0.00 |
| **M138I** | 14.82 | 0.00 | 13.34 | 20.29 | 25.72 | **M138I** | 14.73 | 0.00 | 13.26 | 20.24 | 25.77 |
| **T199I** | 5.00 | 10.92 | 8.62 | 9.90 | 17.09 | **T199I** | 5.00 | 10.81 | 8.54 | 9.88 | 17.06 |
| **L272M** | 11.55 | 12.73 | 17.09 | 13.26 | 0.00 | **L272M** | 10.99 | 12.06 | 16.87 | 12.62 | 0.00 |
| **A80V** | 12.57 | 10.89 | 26.44 | 28.92 | 22.65 | **A80V** | 12.49 | 10.90 | 26.38 | 28.70 | 22.51 |
| **S111R** | 13.89 | 9.78 | 23.69 | 27.96 | 27.70 | **S111R** | 13.79 | 9.76 | 23.53 | 27.72 | 27.62 |
| **A204G** | 6.53 | 15.60 | 8.02 | 0.00 | 12.75 | **A204G** | 6.57 | 15.47 | 8.06 | 0.00 | 12.19 |
| **L130V** | 6.28 | 3.81 | 6.89 | 13.11 | 19.84 | **L130V** | 6.29 | 3.81 | 6.89 | 13.15 | 20.13 |
| **L271F** | 11.80 | 9.77 | 14.37 | 11.70 | 0.00 | **L271F** | 12.23 | 9.10 | 14.79 | 11.83 | 0.00 |
| **A49V** | 14.46 | 23.32 | 25.42 | 26.13 | 22.25 | **A49V** | 14.37 | 23.34 | 25.37 | 26.03 | 21.73 |
| **K77E** | 16.98 | 8.84 | 29.91 | 32.76 | 26.73 | **K77E** | 16.89 | 8.83 | 29.83 | 32.50 | 26.58 |
| **L140M** | 18.85 | 0.00 | 11.30 | 22.48 | 28.62 | **L140M** | 18.81 | 0.00 | 11.19 | 22.52 | 28.71 |
| **I313F** | 14.40 | 5.63 | 21.51 | 20.89 | 8.31 | **I313F** | 14.27 | 5.57 | 21.45 | 20.40 | 7.20 |
| **S137T** | 18.25 | 0.00 | 16.04 | 23.65 | 27.85 | **S137T** | 18.21 | 0.00 | 15.96 | 23.64 | 27.86 |
| **Q180H** | 11.34 | 8.60 | 5.08 | 11.67 | 21.30 | **Q180H** | 11.43 | 8.52 | 5.05 | 11.86 | 21.62 |
| **T45A** | 19.72 | 21.61 | 28.45 | 30.10 | 28.72 | **T45A** | 19.73 | 21.42 | 28.38 | 30.07 | 28.39 |
| **E144V** | 17.16 | 3.80 | 14.37 | 23.55 | 33.44 | **E144V** | 17.19 | 3.80 | 14.35 | 23.74 | 33.76 |
| **M314T** | 12.61 | 8.05 | 22.02 | 22.15 | 10.99 | **M314T** | 12.50 | 7.91 | 21.88 | 21.64 | 10.02 |
| **I341T** | 22.23 | 34.31 | 31.70 | 25.27 | 17.44 | **I341T** | 22.23 | 34.45 | 31.83 | 25.20 | 17.25 |
| **S102T** | 6.00 | 6.91 | 11.17 | 15.00 | 17.32 | **S102T** | 6.08 | 6.94 | 11.08 | 14.80 | 17.52 |
| **V176M** | 11.60 | 11.44 | 0.00 | 9.93 | 20.12 | **V176M** | 11.65 | 11.20 | 0.00 | 10.08 | 20.48 |

**Table S2 Co-evolving amino acid pairs in the L4, L5, L7 loops and the dimer interface (DI).** The raw and scaled scores were calculated by the Gremlin program ^1^. Amino acid pairs with scaled score > 1 were considered to co-evolve. Functional regions are indicated in parenthesis.

| **co-evolving** | | **Gremlin results** | | |
| --- | --- | --- | --- | --- |
| **AA1** | **AA2** | **raw score** | **scaled score** | **probability** |
| 140 (DI) | 152 (DI) | 0.10 | 1.46 | 0.93 |
| 204 (L5) | 207 (L5) | 0.08 | 1.16 | 0.76 |
| 71 (DI) | 137 (DI) | 0.08 | 1.14 | 0.75 |
| 177 (L4) | 207 (L5) | 0.08 | 1.12 | 0.73 |
| 137 (DI) | 269 (L7) | 0.08 | 1.11 | 0.72 |
| 309 (DI) | 261 (L7) | 0.07 | 1.04 | 0.65 |

**Table S3 Structures of evolutionary intermediates.** Number and occupancies of the zinc ions and ligands. Abbreviations CAC: cacodylate, HLN: hexyl(naphthalen-2-yloxy)phosphonic acid. The 4XAZ structure was used to represent the R18 variant.

| **Variant** | **PDB ID** | **Subunit A** | | | **Subunit B** | | |
| --- | --- | --- | --- | --- | --- | --- | --- |
|  |  | **Zn-Zn distance (Å)** | **Zn ions**  **(occupancy)** | **Ligand (occupancy)** | **Zn-Zn distance (Å)** | **Zn ions**  **(occupancy)** | **Ligand (occupancy)** |
| **R0** | **4PCP** | 3.68 | 2  (0.82; 0.84) | CAC  (0.69) | 3.77 | 2  (1.00; 0.86) | CAC  (0.69) |
| **R1** | **4XAF** | 3.87 | 2  (0.60; 0.31) | CAC  (0.69) | 3.78 | 2  (0.67; 0.43) | CAC  (0.69) |
| **R2** | **4XD5** | 3.71 | 2  (1.00; 0.77) | CAC  (0.68) | 3.80 | 2  (0.94; 0.87) | CAC  (0.79) |
| **R6** | **4XAG** | 3.29 | 2  (1.00; 1.00) | - | 3.34 | 2  (1.00; 1.00) | CAC  (0.79) |
| **R8** | **4XAY** | 3.31 | 2  (1.00; 1.00) | - | 3.42 | 2  (1.00; 1.00) | CAC  (0.60) |
| **R18** | **4GY0** | 2.36 | 3  (0.79; 0.33; 0.70) | - | 2.11 | 3  (0.57; 0.36; 0.74) | - |
| **R18** | **4GY1** | 3.58 | 3  (0.80; 0.38; 0.22) | CAC  (0.45) | 1.94 | 3  (0.72; 0.36; 0.36) | CAC  (0.58) |
| **R18** | **4E3T** | 3.84 | 3  (0.67; 0.50; 0.31) | - | 3.87 | 3  (0.60; 0.47; 0.46) | HLN  (0.68) |
| **R18** | **4XAZ** | 3.31 | 2  (1.00; 1.00) | - | 3.31 | 2  (1.00; 0.95) | - |
| **R22** | **4PCN** | 3.27 | 2  (1.00; 0.98) | - | 3.23 | 2  (0.86; 0.95) | - |

**Table S4 Loop distances and Zn-Zn separation in different crystal structures.** The **L5** – **L7** distance was measured between A/G204 Cα – G273 Cα, while the **L4** – **L5** distance between G174 Cα – S205 Cα. Distances are given in Å.

| **Variant** | **PDB** | **A subunit** | | | **B/G subunit** | | |
| --- | --- | --- | --- | --- | --- | --- | --- |
|  |  | **d_L4-L5_** | **d_L5-L7_** | **d_Zn-Zn_** | **d_L4-l5_** | **d_L5-L7_** | **d_Zn-Zn_** |
| **R0** | 4PCP | 8.41 | 12.75 | 3.68 | 8.37 | 12.19 | 3.77 |
| **R1** | 4XAF | 9.51 | 13.28 | 3.87 | 8.44 | 12.21 | 3.78 |
| **R2** | 4XD5 | 9.72 | 13.09 | 3.71 | 8.42 | 12.64 | 3.80 |
| **R6** | 4XAG | 7.45 | 13.64 | 3.29 | 10.94 | 7.07 | 3.34 |
| **R8** | 4XAY | 7.37 | 13.42 | 3.31 | 10.28 | 7.06 | 3.42 |
| **R18** | 4GY0 | 6.12 | 11.76 | 2.36 | 9.32 | 9.22 | 2.11 |
| **R18** | 4GY1 | 6.05 | 10.98 | 3.58 | 9.76 | 8.46 | 1.94 |
| **R18** | 4E3T | 6.56 | 11.24 | 3.84 | 9.21 | 9.04 | 3.87 |
| **R18** | 4XAZ | 6.98 | 12.17 | 3.31 | 8.55 | 8.43 | 3.31 |
| **R22** | 4PCN | 4.70 | 11.97 | 3.27 | 9.42 | 9.27 | 3.23 |

**Table S5** (separate xls) **PTE evolutionary intermediates in different organisms.** Representative loop distances, mutations, kinetic data for different activities and the references are displayed.

**Figure S1 Asymmetric coupling between changes in dynamics and structure in individual subunits during the PTE → AE trajectory. (A)-(B)** Structure-dynamics covariance during the emergence (A) and specialisation (B) for the new function in the A subunit. **(C)-(D)** Structure-dynamics covariance during the emergence (C) and specialisation (D) for the new function in the B subunit. Covariance was computed from deviations in disorder scores and Cα atom positions from the starting R0 variant as defined by eq (6). The loops (L4 lime, L5 red, L7 blue) and dimer interface (D, orange) are shown on the side panels.

**Figure S2 Divergence of loop conformations during the PTE → AE evolution.** Deviations between the two subunits in distances from the R0 variant were averaged for the Cα atoms of the **L4** (green), **L5** (red), **L7** (blue) loops (Δd_A-B_). The error bars show the residue-based variations.


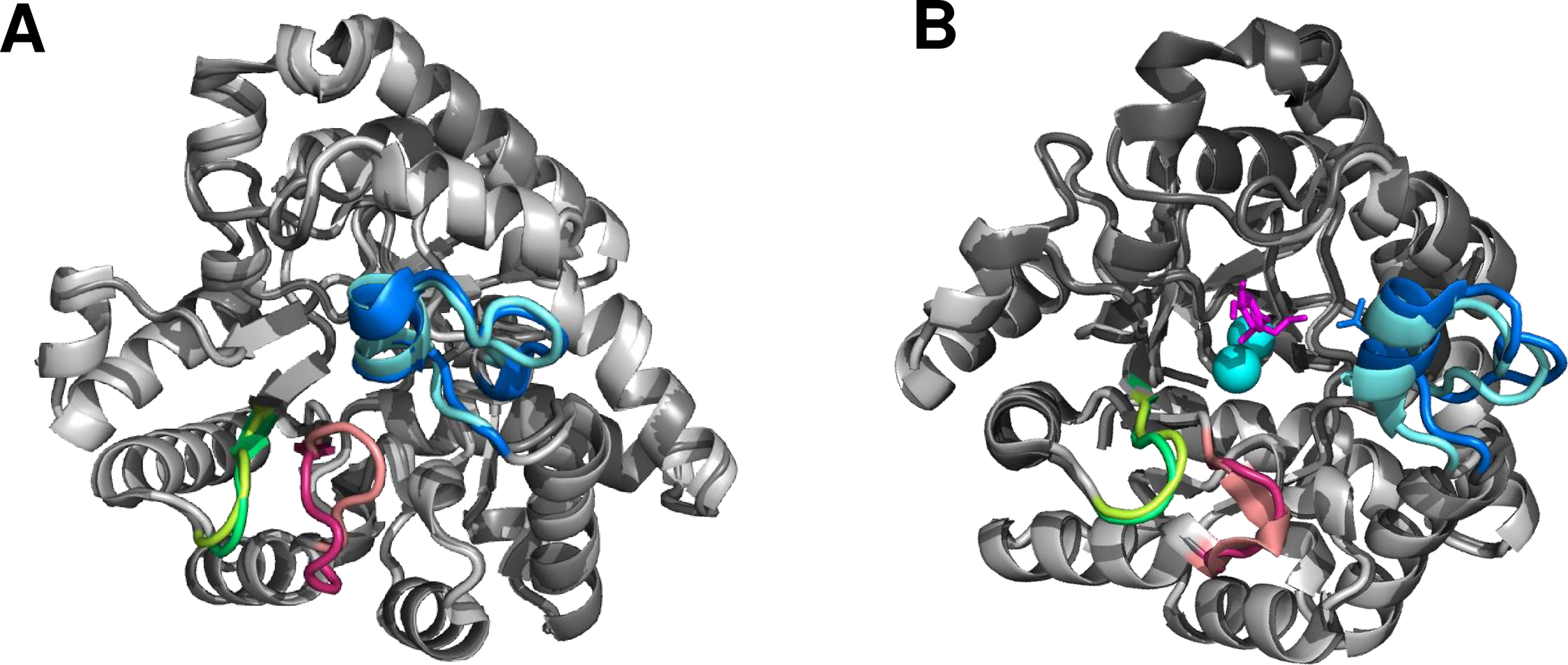


**Figure S3 Structural comparison of the loops (A) in the two subunits in the R8 generalist state (PDB: 4xay); (B) in the closed (PDB:2r1n) and open (3a3w) states. (A)** The loops are colored as L4 - green, L5 - darkpink, L7 - marine In A subunit of the R8 generalist state (PDB: 4xay), and L4 - limon, L5 - salmon, L7 - cyan in the B subunit of the R8 generalist state (PDB: 4xay). **(B)** The loops are colored as L4 - green, L5 - darkpink, L7 - marine in the closed state (PDB:2r1n), and L4 - limon, L5 - salmon, L7 - cyan in the open state (PDB:3a3w).

The L4-L5 and L5-L7 distances markedly differ from each other in the two subunits of the R8 generalist state: d_L4-L5_ (A) = 10.3 Å**,** d_L4-L5_ (B) = 7.4 Å; d_L5-L7_ (A)= 13.4 Å , d_L5-L7_ (B)= 7.1 Å **(A)**, while they are more similar in the previously characterised open and closed states: d_L4-L5_ (closed) = 8.5 Å**,** d_L4-L5_ (open) = 9.3 Å; d_L5-L7_ (closed)= 11.1 Å , d_L5-L7_ (open)= 15.0 Å **(B)**.

**References**

1 Kamisetty, H., Ovchinnikov, S. & Baker, D. Assessing the utility of coevolution-based residue-residue contact predictions in a sequence- and structure-rich era. *Proc Natl Acad Sci U S A* **110**, 15674-15679, doi:10.1073/pnas.1314045110 (2013).
